# Diverse Microvirus Genomes Identified in the Stomach of a Sharp-Spined Notothen (Trematomus pennellii) from the Ross Sea (East Antarctica)

**DOI:** 10.1128/mra.01233-22

**Published:** 2023-01-26

**Authors:** Jasmine K. M. Lopez, Charlotte Austin, Kata Farkas, Simona Kraberger, William Davison, Arvind Varsani

**Affiliations:** a The Biodesign Center for Fundamental and Applied Microbiomics, Center for Evolution and Medicine and School of Life Sciences, Arizona State University, Tempe, Arizona, USA; b School of Biological Sciences, University of Canterbury, Christchurch, New Zealand; c School of Natural Sciences, Bangor University, Bangor, United Kingdom; d Structural Biology Research Unit, Department of Integrative Biomedical Sciences, University of Cape Town, Cape Town, South Africa; Queens College Department of Biology

## Abstract

Sharp-spined notothen (Trematomus pennellii) is an icefish endemic to the southern ocean. From the stomach of an individual, we identified the genomes of 51 microviruses (family *Microviridae*). The major capsid proteins of most of these share the closest similarities to those identified in other marine organisms.

## ANNOUNCEMENT

The sharp-spined notothen (Trematomus pennellii) is a member of the Nototheniidae family and endemic to the southern ocean (1). There is limited information on viruses associated with Antarctic fish in general. Prior research on Antarctic fish identified a papillomavirus in emerald notothen (Trematomus bernacchii) (2) and two polyomaviruses in an emerald notothen and a sharp-spined notothen (3, 4).

A sharp-spined notothen was caught in the McMurdo Sound (Ross Sea, Antarctica) using traditional hook and line fishing during the 2012–2013 austral summer. The fish was caught under the 2011/08R animal ethics permit (University of Canterbury, New Zealand). Approximately 0.5 cm^3^ of stomach was dissected and homogenized in 20 mL of SM buffer (0.1 M NaCl, 50 mM Tris-HCl, pH 7.4, 10 mM MgSO_4_) with mortar and pestle. The homogenate was centrifuged at 6,000 × *g* for 10 min to pellet cell debris. The supernatant was sequentially filtered through 0.45- and 0.22-μm syringe filters, and viral particles in the filtrate were precipitated with 15% (wt/vol) polyethylene glycol 8000 (PEG 8000). The resulting solution was centrifuged at 6,000 × *g* for 20 min, and the pellet was resuspended in 1 mL of SM buffer. Viral DNA was extracted from 200 μL solution using the High Pure viral nucleic acid kit (Roche Diagnostics, Germany). Circular DNA was preferentially amplified using rolling circle amplification (RCA) with a TempliPhi 100 kit (GE Healthcare, USA). A library using the TruSeq Nano DNA kit (Illumina, USA) was prepared from the RCA products and sequenced on an Illumina HiSeq 4000 sequencer at Macrogen Inc. (Korea). The sequence reads (23,631,809 paired-end reads; average read length, 101 nucleotides [nt]) were quality trimmed using Trimmomatic v0.39 (5) and then *de novo* assembled using metaSPAdes v3.14 (6). Circular genomes were identified based on terminal redundancy. All bioinformatic tools were run with default parameters.

**FIG 1:**
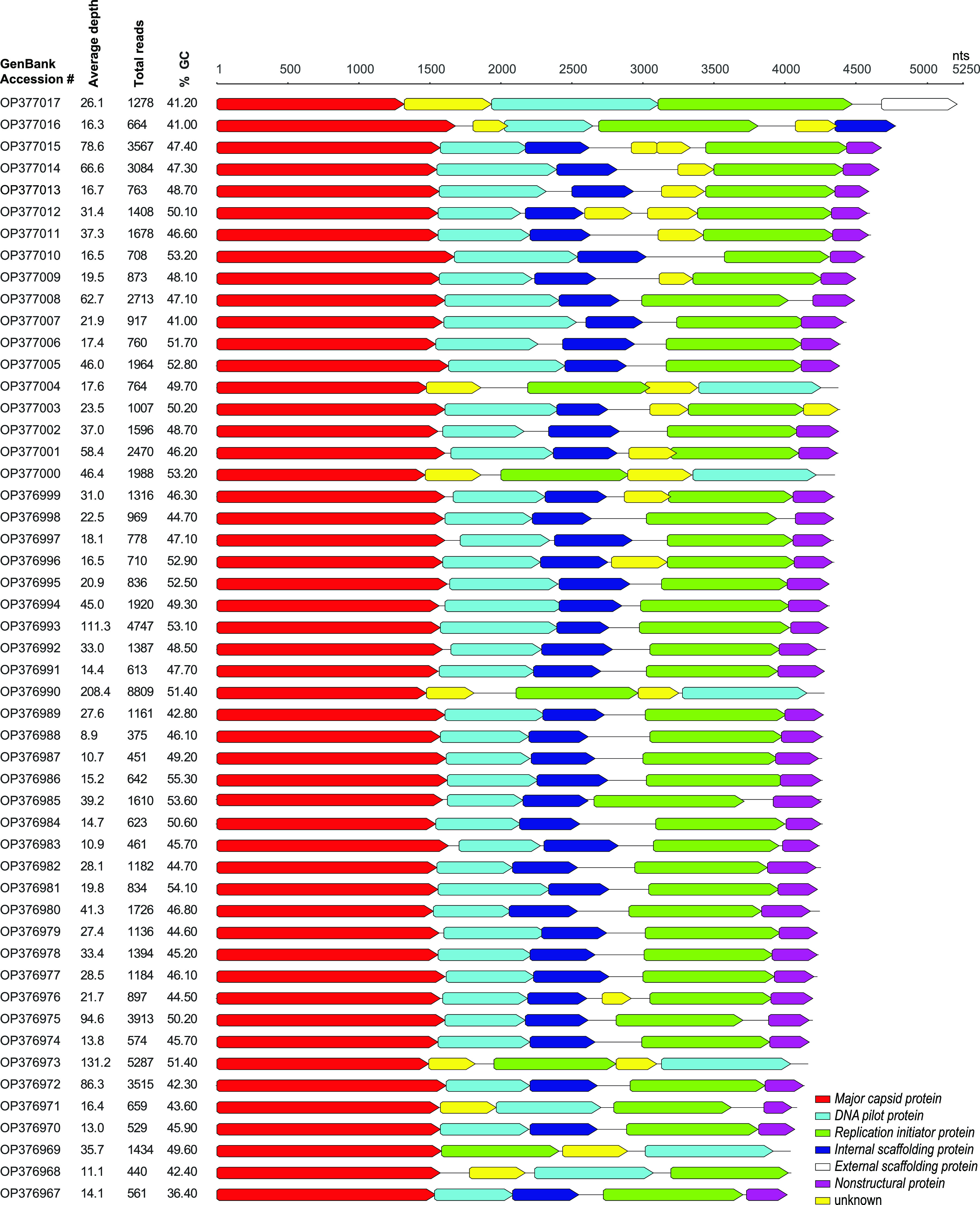
Genome organization of the 51 microviruses identified in the stomach of a sharp-spined notothen caught in the Ross Sea in east Antarctica. A summary of the GC%, read depth, and number of reads is provided for each genome.

Circular contigs (>1,000 nt in length) were analyzed using BLASTx (7) for viral-like sequences using a RefSeq viral protein database (RefSeq release 207). We identified 51 circular contigs (4,012 to 5,250 nt) with similarities to the microvirus proteins (family *Microviridae*). Microviruses are small circular single-stranded DNA bacteriophages in the order *Petitvirales* and phylum *Phixviricota* (8). These 51 microvirus genomes have coverage depth of 8.9× to 280.4×, number of mapped reads of 375 to 8,809, and GC content of 36.4 to 55.3% (Fig. 1). VIBRANT (9) was used to annotate the genomes with additional verification with BLASTp (7) similarity searches. Of these genomes, 82% have a similar organization of the relatively conserved open reading frames, i.e., major capsid protein, DNA pilot protein, replication initiator protein, internal scaffolding protein, and nonstructural protein (Fig. 1). The major capsid protein is the most conserved protein in all microviruses, and BLASTp analysis revealed that majority shared similarities with sequences of microviruses of marine organisms, i.e., abalone tissue (*n* = 1), crucian tissue (*n* = 1), red snapper tissue (*n* = 1), haddock tissue (*n* = 4), minnow tissue (*n* = 5), and sea quirt (*n* = 30), while there was a small number shared with those from cold methane seep sediment (*n* = 1), robin feces (*n* = 1), human gut sample (*n* = 1), lake water sample (*n* = 1), and sewage oxidation pond, sludge, and wastewater (*n* = 5) (Table 1) (10–15). The BLASTp hits ranged from 41% to 83% (Table 1). It is highly likely that the 51 microviruses infect gut-associated bacteria of the sharp-spined notothen or their diet.

**TABLE 1:** Summary of the top BLASTx hit for the MCP of the 51 microviruses described in this study

Query sequence (GenBank accession no.)	Data for major capsid protein top BLAST hit			
	Best hit MCP (GenBank accession no.)	Virus	Microvirus source	% identity
OP376967	MH572390	*Microviridae* sp. strain SD_SF_42	Ciona robusta intestinal tract	83.3
OP376968	MH572372	*Microviridae* sp. strain SD_SC_58	Ciona robusta intestinal tract	56.4
OP376969	MH572461	*Microviridae* sp. strain SD_MC_53	Ciona robusta intestinal tract	56.4
OP376970	MH572325	*Microviridae* sp. strain SD_SC_10	Ciona robusta intestinal tract	72.3
OP376971	MT310128	*Microviridae* sp. strain 6424_116	Wastewater	57.3
OP376972	MH572453	*Microviridae* sp. strain SD_MC_6	Ciona robusta intestinal tract	72.0
OP376973	MH572365	*Microviridae* sp. strain SD_SC_34	Ciona robusta intestinal tract	68.5
OP376974	MH616998	*Microviridae* sp. strain ctdc857	Minnow tissue	61.1
OP376975	MH617576	*Microviridae* sp. strain ctcc822	Minnow tissue	58.4
OP376976	MH572289	*Microviridae* sp. strain SD_SC_80	Ciona robusta intestinal tract	74.1
OP376977	MH572441	*Microviridae* sp. strain SD_MC_80	Ciona robusta intestinal tract	66.4
OP376978	MH617039	*Microviridae* sp. strain ctda820	Minnow tissue	66.2
OP376979	MH572493	*Microviridae* sp. strain SD_HF_33	Ciona robusta intestinal tract	61.4
OP376980	MT310213	*Microvirus* sp. strain 1712115_898	Sludge	63.7
OP376981	MH616888	*Microviridae* sp. strain cthi64	Haddock tissue	63.2
OP376982	MH617651	*Microviridae* sp. strain ctcc35	Red snapper tissue	63.0
OP376983	MH572299	*Microviridae* sp. strain SD_SC_76	Ciona robusta intestinal tract	66.0
OP376984	MT310091	*Microviridae* sp. strain 6434_67	Wastewater	64.2
OP376985	MT309961	*Microviridae* sp. strain BS1_412	Sewage oxidation pond	60.2
OP376986	MH572474	*Microviridae* sp. strain SD_MF_21	Ciona robusta intestinal tract	62.5
OP376987	MH572345	*Microviridae* sp. strain SD_SC_53	Ciona robusta intestinal tract	65.0
OP376988	MZ364287	Robinz microvirus RP_160	Feces of robin	67.4
OP376989	MH572299	*Microviridae* sp. strain SD_SC_76	Ciona robusta intestinal tract	72.7
OP376990	MH572365	*Microviridae* sp. strain SD_SC_34	Ciona robusta intestinal tract	67.9
OP376991	MT310030	*Microviridae* sp. strain 6538_71	Wastewater	61.3
OP376992	MH572501	*Microviridae* sp. strain SD_HF_34	Ciona robusta intestinal tract	66.0
OP376993	MH616888	*Microviridae* sp. strain cthi64	Haddock tissue	69.6
OP376994	MH616888	*Microviridae* sp. strain cthi64	Haddock tissue	70.9
OP376995	MH552548	*Microviridae* sp. strain ctjb11	Abalone tissue	57.8
OP376996	MH572501	*Microviridae* sp. strain SD_HF_34	Ciona robusta intestinal tract	63.8
OP376997	MH572501	*Microviridae* sp. strain SD_HF_34	Ciona robusta intestinal tract	66.9
OP376998	MH572299	*Microviridae* sp. strain SD_SC_76	Ciona robusta intestinal tract	72.9
OP376999	MH572493	*Microviridae* sp. strain SD_HF_33	Ciona robusta intestinal tract	61.8
OP377000	MH572365	*Microviridae* sp. strain SD_SC_34	Ciona robusta intestinal tract	68.8
OP377001	MH572289	*Microviridae* sp. strain SD_SC_80	Ciona robusta intestinal tract	59.3
OP377002	MH622906	*Microviridae* sp. strain ctbj815	Minnow tissue	65.0
OP377003	MH617086	*Microviridae* sp. strain ctcc904	Minnow tissue	64.4
OP377004	MH572365	*Microviridae* sp. strain SD_SC_34	Ciona robusta intestinal tract	67.9
OP377005	MH616888	*Microviridae* sp. strain cthi64	Haddock tissue	65.2
OP377006	MK012456	*Microviridae* sp. strain ctcb6	Crucian tissue	63.4
OP377007	MW697696	Arizlama microvirus AZLM_250	Lake water sample	68.3
OP377008	MH572481	*Microviridae* sp. strain SD_MF_9	Ciona robusta intestinal tract	73.4
OP377009	MH572451	*Microviridae* sp. strain SD_MC_7	Ciona robusta intestinal tract	73.2
OP377010	MH572325	*Microviridae* sp. strain SD_SC_10	Ciona robusta intestinal tract	67.9
OP377011	MH572451	*Microviridae* sp. strain SD_MC_7	Ciona robusta intestinal tract	71.5
OP377012	MH572337	*Microviridae* sp. strain SD_SC_2	Ciona robusta intestinal tract	66.9
OP377013	MH572451	*Microviridae* sp. strain SD_MC_7	Ciona robusta intestinal tract	64.4
OP377014	MH572451	*Microviridae* sp. strain SD_MC_7	Ciona robusta intestinal tract	67.4
OP377015	MH572451	*Microviridae* sp. strain SD_MC_7	Ciona robusta intestinal tract	72.1
OP377016	BK051953	*Microviridae* sp. strain cttI53	Human metagenome	53.7
OP377017	KP087943	Eel River Basin pequenovirus	Cold methane seep sediment	41.4

### Data availability.

The microvirus sequences have been deposited in NCBI databases under BioProject accession number PRJNA874327, BioSample accession number SAMN30543052, SRA accession number SRR21284442, and GenBank accession numbers OP376967 to OP377017.
